# Projektbericht zur Förderung wissenschaftlicher Kompetenzen im Fach Schmerzmedizin im Rahmen der curricularen Lehre

**DOI:** 10.1007/s00482-022-00628-y

**Published:** 2022-03-04

**Authors:** Martin Dusch, Manfred Mayer, Rolf-Detlef Treede, Martin R. Fischer, Markus Berndt

**Affiliations:** 1 Klinik für Anästhesiologie und Intensivmedizin, Medizinische Hochschule Hannover, Carl-Neuberg-Str. 1, 30625 Hannover, Deutschland; 2 Mannheim Institute of Public Health, Medizinische Fakultät Mannheim, Ruprecht-Karls-Universität Heidelberg, Mannheim, Deutschland; 3 Centrum für Biomedizin und Medizintechnik Mannheim, Medizinische Fakultät Mannheim, Ruprecht-Karls-Universität Heidelberg, Mannheim, Deutschland; 4 Institut für Didaktik und Ausbildungsforschung in der Medizin, Klinikum der Universität München, LMU München, München, Deutschland

**Keywords:** Kompetenzbasierte Lehre, Arztrolle des Gelehrten, Wissenschaftsmodul, NKLM, Competence-based teaching, Scholar, Research module, NKLM

## Abstract

**Zielsetzung:**

Die Vermittlung von Wissenschaftskompetenzen ist ein unverzichtbares Charakteristikum eines akademischen Medizinstudiums. Im Rahmen des Modellstudiengangs Mannheimer Reformiertes Curriculum für Medizin (MaReCuM) wurde die Lehrveranstaltung *Wissenschaftliches Arbeiten im Fach Schmerzmedizin *in das 5. Studienjahr implementiert. Die Ziele der Arbeit umfassen die Beschreibung dieser kompetenzorientierten Lehrveranstaltung sowie eine Evaluation der Lerneffekte der Lehrveranstaltung.

**Methodik:**

114 Studierende haben sich an einer Fragebogenerhebung beteiligt. Die historische Kontrollgruppe absolvierte das 5. Studienjahr vor Einführung der Lehrveranstaltung. Die Interventionsgruppe nahm verpflichtend an der Lehrveranstaltung sowie an der realen Versorgungsforschungsstudie *Case-Management-Programm Kreuzschmerz*teil. In beiden Gruppen erfolgte eine Fragebogenerhebung zu schmerzmedizinischem Vorwissen und Interesse sowie zur Akzeptanz der Lehrveranstaltung und dem subjektiv wahrgenommenen Lernerfolg.

**Ergebnisse:**

Die innovative und kompetenzorientierte Lehrveranstaltung konnte erfolgreich in das Curriculum des Modellstudiengangs implementiert und mit den Partnern in der Allgemeinmedizin und dem Mannheim Institute of Public Health wie geplant durchgeführt werden. Die Lehrveranstaltung wurde von den teilnehmenden Studierenden akzeptiert. In der begleitenden Evaluation hatte die Teilnahme an der Lehrintervention an und für sich keinen messbaren Einfluss auf den *subjektiven* Lernerfolg.

**Diskussion:**

Unseres Wissens nach wurde dieser didaktische Ansatz in der curricularen Lehre bislang noch nicht verfolgt. Die vorgestellte Lehrveranstaltung eröffnet eine weitere Option zur Vermittlung von Wissenschaftskompetenzen im Rahmen des Medizinstudiums. Ein Effekt der Lehrveranstaltung auf den subjektiven Lernerfolg war in der untersuchten Form und am Ende des Moduls nicht messbar. Gründe dafür könnten in den vielfältigen und umfangreichen Vorerfahrungen der Studierenden des Modellstudiengangs MaReCuM sowie in Limitationen bei der Teilnahme an der realen Versorgungsforschungsstudie liegen. Durch die Verknüpfung der Lehrintervention mit anderen Lehrveranstaltungen zu einem longitudinalen Wissenschaftsmodul kann eine zusätzliche Lerngelegenheit im Bereich der Arztrolle des Gelehrten geschaffen werden. Die Implementierung der Lehrveranstaltung bietet darüber hinaus die Gelegenheit für vergleichende Untersuchungen zum Erwerb von Wissenschaftskompetenzen der Studierenden im Fach Humanmedizin.

## Abstract (en)

**Aim:**

The training of scientific skills and competencies is an essential part of academic medical studies. As part of the MaReCuM model study program at Heidelberg University’s Mannheim Medical School, a fifth-year rotation on scientific skills in the field of pain medicine was implemented. This paper describes this competence-oriented rotation as well as the investigation of the educational effect.

**Method:**

A total of 114 fifth-year medical students participated in the survey (response rate: 83%). The control group completed the fifth year prior to the implementation of the rotation. The experimental group was required to participate in the rotation and the real healthcare research study “Case management program: low back pain”. A survey of both groups was conducted on the first day of the rotation and at the end of the module.

**Results:**

The innovative and competency-based learning unit was successfully implemented as part of the MaReCuM model study program and carried out with partners in general practice as well as the Mannheim Institute of Public Health. The participating students accepted the rotation well. There was no measurable effect on the subjective learning success of the rotation in the evaluation.

**Discussion:**

To the authorsʼ knowledge, this educational approach has never been tested before in a German study program. The presented rotation offers an additional option for the training of scientific competencies as part of medical studies. The missing of a measurable effect could be due to the extensive experience of the medical students as well as the limitations on participation in a real healthcare study. An additional learning opportunity could be created by connecting the preexisting lectures to a longitudinal module on scholarly competencies. The implementation of the program also offers a unique opportunity for educational research on the acquisition of scientific competencies in medical students.

## Einleitung

Der Arztberuf verpflichtet zu einem lebenslangen Lernen, Anwenden und Weitergeben von medizinischem Wissen. Die Erweiterung des medizinischen Wissens sowie die kritische Auseinandersetzung mit neuen Erkenntnissen gehören ebenfalls zu den ärztlichen Aufgaben [1]. Dennoch ist die Arztrolle des Gelehrten in medizinischen Curricula verbreitet unterrepräsentiert [2–5]. Ärztinnen und Ärzte in Weiterbildung geben als Barrieren für eine akademische Entwicklung fehlende Fertigkeiten und einen Mangel an formaler Ausbildung an [6]. Die Empfehlungen des Wissenschaftsrats zur Weiterentwicklung des Medizinstudiums in Deutschland aus dem Jahr 2014 setzten daher einen Schwerpunkt in der Stärkung der wissenschaftlichen Kompetenzen der Studierenden [7]. Gefordert wurden insbesondere Kompetenzen, die für das Verstehen, Anwenden und Bewerten wissenschaftlicher Konzepte und Methoden nötig sind. So sollte deren Transfer in die klinische Praxis im Rahmen der Patientenversorgung sowie in das wissenschaftliche Arbeiten ermöglicht werden. Vor dem Hintergrund einer evidenzbasierten Medizin sind diese Kompetenzen für alle Ärztinnen und Ärzte erforderlich, unabhängig davon, ob eine explizite wissenschaftliche Karriere angestrebt wird oder nicht [7, 8]. Mit dem Nationalen Kompetenzbasierten Lernzielkatalog Medizin (NKLM) wurde ein Rahmen für die erforderlichen Kompetenzen geschaffen [9, 10]. Um die sehr unterschiedlichen oben genannten akademischen Anforderungen an eine Ärztin und einen Arzt abzubilden, bündelt die Arztrolle des Gelehrten sowohl im NKLM als auch in vergleichbaren internationalen Rahmenwerken sehr heterogene Kompetenzen [11]. Im Gegensatz zu den vergleichbaren internationalen Rahmenwerken legt der NKLM sowohl quantitativ als auch bezüglich der qualitativen Differenziertheit den Schwerpunkt auf Kompetenzen, die Ärztinnen und Ärzte befähigen sollen, selbst in der Forschung zu arbeiten [11].

Vor diesem Hintergrund wurde die Lehrveranstaltung *Wissenschaftliches Arbeiten im Fach Schmerzmedizin *in den Modellstudiengang Mannheimer Reformiertes Curriculum für Medizin (MaReCuM) der medizinischen Fakultät Mannheim der Universität Heidelberg eingeführt. Ziel war es, die Studierenden durch die Teilnahme an einem realen Forschungsprojekt im Rahmen der curricularen Lehre auf Aspekte des wissenschaftlichen Arbeitens vorzubereiten. Durch den unmittelbaren Praxisbezug sollte wissenschaftliches Arbeiten im Rahmen der curricularen Lehre erlebbar werden. Unseres Wissens nach wurde dieser didaktische Ansatz bislang noch nicht verfolgt. In diesem Artikel beschreiben und evaluieren wir dieses innovative und kompetenzorientierte Lehrformat. Nach der Implementierung werden sich modellhaft Untersuchungen zum Erwerb von Wissenschaftskompetenzen durchführen lassen.

## Projektbeschreibung

### Lernziele der Lehrveranstaltung *Wissenschaftliches Arbeiten im Fach Schmerzmedizin*

Für die Lehrveranstaltung *Wissenschaftliches Arbeiten im Fach Schmerzmedizin* wurden acht Lernziele für die Studierenden formuliert. Diese wurden in Anlehnung an die übergeordneten Lernziele des Kapitels 6 „Die Ärztin und der Arzt als Gelehrte/-r“ des NKLM [10] formuliert oder sind diesem entnommen:

1. Sie können Qualitätskriterien wissenschaftlichen Arbeitens benennen und erkennen sowie Störgrößen benennen.
2. Sie können methodenkritische Kenntnisse bei der Planung und Auswertung wissenschaftlicher Studien nutzen.
3. Sie können das eigene Handeln methodenkritisch hinterfragen.
4. Sie können sich an ethischen Normen guter wissenschaftlicher Praxis orientieren.
5. Sie können sich die notwendigen wissenschaftlichen Informationen zur Beantwortung einer medizinischen Frage beschaffen.
6. Sie können Ergebnisse generieren, analysieren und darstellen.
7. Sie können Grundlagen der Studien- und Versuchsplanung benennen.
8. Sie können Fragebogen als Anamnesetool einsetzen und auswerten.

### Aufbau der Lehrveranstaltung

Die Lehrveranstaltung *Wissenschaftliches Arbeiten im Fach Schmerzmedizin* wurde im 5. Studienjahr in das Modul Primärversorgung implementiert und bestand aus einem Seminar mit 2 Unterrichtseinheiten (UE à 45 min), einem Kleingruppenunterricht mit einer UE sowie 2 jeweils einstündigen Untersuchungsterminen im Rahmen des Blockpraktikums Allgemeinmedizin. Während dieser Termine sollten die Studierenden reale Studienpatienten der Versorgungsforschungsstudie *Case-Management-Programm Kreuzschmerz* untersuchen. In der Seminardoppelstunde wurden das Projekt, an dem die Studierenden aktiv forschend teilnahmen, sowie dessen Ablauf vorgestellt. Dabei wurde der logistische Ablauf der Datenerhebung besprochen, die Studierenden wurden in die teilnehmenden Lehrpraxen eingeteilt. Im Kleingruppenunterricht wurden das Einsetzen von Fragebögen in der Wissenschaft und die in dem Forschungsprojekt konkret eingesetzten Fragebögen (paindetect, SES, SF12, START und Schmerzzeichnung) besprochen. Dazu füllten die Studierenden die Fragebögen selbst aus und werteten diese unter Anleitung im Anschluss aus. So wurden mögliche Fehlerquellen identifiziert und Strategien zur Fehlervermeidung besprochen. Im Anschluss wurden die in der Hausarztpraxis durchzuführenden klinischen Untersuchungen demonstriert und die Regeln für eine einheitliche schriftliche Dokumentation der Untersuchungsergebnisse erklärt.

### Training der Praxisinhaber der am Blockpraktikum Allgemeinmedizin teilnehmenden Praxen und Ablauf der Studienteilnahme

**Figure 1:**
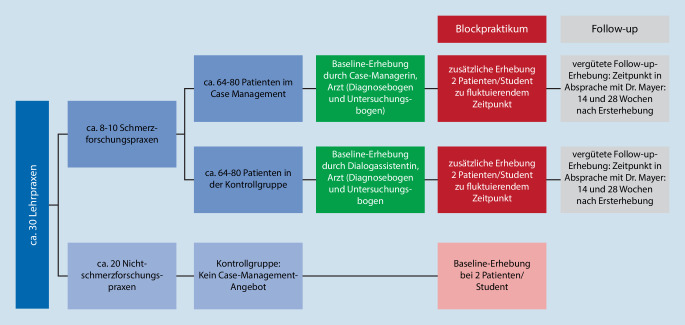

Für das Blockpraktikum Allgemeinmedizin standen sowohl Allgemeinmedizinpraxen, die an der Versorgungsforschungsstudie *Case-Management-Programm Kreuzschmerz* teilnahmen (Schmerzforschungspraxen), als auch Allgemeinmedizinpraxen, die nicht an dieser Studie teilnahmen (Nichtschmerzforschungspraxen), zur Verfügung. Die Praxisinhaber aller am Blockpraktikum Allgemeinmedizin teilnehmenden Praxen wurden sowohl über die Versorgungsforschungsstudie als auch die vorliegende Lehrforschungsstudie informiert. Alle Praxisinhaber erklärten sich bereit, den Studierenden im Rahmen des 14-tägigen Blockpraktikums Allgemeinmedizin zwei Patienten mit Kreuzschmerzen einzubestellen. Die Patienten der Schmerzforschungspraxen waren zum Zeitpunkt der Untersuchung durch die Studierenden bereits für einen der zwei Studienarme randomisiert (entweder Teilnahme am *Case-Management-Programm Kreuzschmerz* oder Teilnahme an der Kontrollgruppe). Die Patienten der Nichtschmerzforschungspraxen wurden am Tag der Untersuchung durch die Studierenden vom Praxisinhaber für die Studienteilnahme aufgeklärt und dem Studienarm Kontrollgruppe ohne Case-Management-Programm-Angebot zugeordnet (Abb. 1). Die Studierenden gaben an die rekrutierten Patienten die oben genannten Schmerzfragebögen aus und werteten diese im Anschluss aus. Darüber hinaus führten sie unter Supervision durch den jeweiligen Praxisinhaber die oben genannten klinischen Untersuchungen an den rekrutierten Patienten durch und füllten einen Diagnosebogen aus. Die Studierenden übertrugen die Untersuchungs- und Fragebogenergebnisse sowie den Diagnosebogen in ein Scoresheet zur weiteren Verarbeitung im Rahmen der Versorgungsforschungsstudie *Case-Management-Programm Kreuzschmerz*.

### Projektevaluation

Das kompetenzorientierte Lehrformat *Wissenschaftliches Arbeiten im Fach Schmerzmedizin* wurde am Ende des Moduls durch die Teilnehmer evaluiert. Untersucht wurde die Frage, ob die Teilnahme am Lehrformat *Wissenschaftliches Arbeiten im Fach Schmerzmedizin *einen Einfluss auf den subjektiv wahrgenommenen Lernerfolg bezüglich der oben genannten Lernziele hat. Dabei wurde von der Annahme ausgegangen, dass Studierende, die am Lehrformat *Wissenschaftliches Arbeiten im Fach Schmerzmedizin* teilgenommen haben, ihren subjektiven Lernerfolg signifikant höher einschätzen als Studierende, die nicht teilgenommen haben. Untersucht wurde ferner die Akzeptanz der Lehrveranstaltung sowie die Frage, ob ein Zusammenhang zwischen dem subjektiven Lernerfolg und der Akzeptanz der Lehrveranstaltung besteht. Dabei wurde von der Annahme ausgegangen, dass ein signifikanter, positiver Zusammenhang zwischen dem subjektiven Lernerfolg und der Akzeptanz der Lehrveranstaltung besteht.

## Methoden

### Design und Stichprobe

Das Projekt wurde mit insgesamt *n* = 114 Studierenden der Humanmedizin im randomisierten Versuchs-Kontrollgruppen-Design durchgeführt. Die Kontrollgruppe (KG) umfasste Studierende im 5. Studienjahr vor der Umsetzung der Einführung des kompetenzorientierten Lehrformats *Wissenschaftliches Arbeiten im Fach Schmerzmedizin* und wurde aus der Studierendenkohorte der Lehrblöcke 3 und 4 im Studienjahr 2013/14 rekrutiert. Für die Interventionsgruppe nahmen Studierende im 5. Studienjahr nach Einführung des verpflichtenden kompetenzorientierten Lehrformats *Wissenschaftliches Arbeiten im Fach Schmerzmedizin* der Lehrblöcke 5 und 6 im Studienjahr 2013/14 sowie des Lehrblocks 1 im Studienjahr 2014/15 teil. In der Kontrollgruppe beteiligten sich 56 Teilnehmer in den beiden Modulen Primärversorgung vor der Einführung des Lehrformats (26 weiblich und 26 männlich, 4 ohne Angabe des Geschlechts; mittleres Alter 26 Jahre). In der Interventionsgruppe (IG) mit verpflichtender Teilnahme am Lehrformat *Wissenschaftliches Arbeiten im Fach Schmerzmedizin* als Teil des Moduls Primärversorgung waren es 58 Teilnehmer (21 weiblich und 30 männlich, 7 ohne Angabe; mittleres Alter 26 Jahre). Neben der Beteiligung an der Befragung ist auch die Beteiligung an der Versorgungsforschungsstudie *Case-Management-Programm Kreuzschmerz* relevant. Die Daten sind in Tab. 1 dargestellt.

**Table 1:** 

	Lehrblock (LB)		
	LB5	LB6	LB1
Anzahl Studierende	28	11	29
Anzahl der Studierenden, die Unterlagen abgegeben haben	18	6	5
Anzahl der Studierenden, die sich gemeldet haben, aber nichts abgegeben haben	6	5	12
Anzahl der Studierenden, von denen keine Rückmeldung kam	4	0	12
Erwartete Fragebogensets	56	22	58
Vollständig abgegebene Fragebogensets	23	12	11
*Rücklaufquote Fragebogensets*	41 %	55 %	19 %
*Beteiligungsrate Studierende (Rücklauf)*	64 %	55 %	17 %
*Beteiligungsrate Studierende (Rückmeldung)*	86 %	100 %	59 %
*Anzahl Fragebögen aus Schmerzpraxen*	6	4	1
Zusätzlich Fragebogensets durch Studierende (Follow-up)	0	10	0
*Gründe Nichtteilnahme*			
Keine geeigneten Patienten	1	4	4
Aus zeitlichen Gründen nicht möglich	1	0	3
Praxis weigerte sich	3	1	4
Keine Teilnahme, da freiwillig	1	0	1

Von den insgesamt *n* = 114 Studierenden befanden sich *n* = 78 in einem Promotionsverfahren oder hatten eine abgeschlossene Promotionsarbeit (*n* = 25 hatten kein Promotionsverfahren eröffnet, *n* = 11 ohne Angaben), *n* = 34 Studierende hatten eine Anstellung als Hilfswissenschaftler (*n* = 68 hatten keine Anstellung als Hilfswissenschaftler, *n* = 12 ohne Angaben), *n* = 16 waren unabhängig von einem Promotionsverfahren an Forschungsprojekten beteiligt (*n* = 87 ohne sonstige Beteiligung an einem Forschungsprojekt, *n* = 11 ohne Angaben), *n* = 5 verfügten über ein abgeschlossenes Erststudium (*n* = 98 ohne abgeschlossenes Erststudium, *n* = 11 ohne Angaben).

### Material

Für die Bewertung des Lehrformats *Wissenschaftliches Arbeiten im Fach Schmerzmedizin* wurden ein Fragebogen zur Prämessung vor der Lehrintervention und ein weiterer Fragebogen zur Postmessung nach der Lehrintervention erstellt. Beide Fragebögen enthielten jeweils mehrere Fragenblöcke mit 6‑stufigen Likert-Items (1 „stimmt überhaupt nicht“ bis 6 „stimmt genau“). Der Aufbau dieser beiden Fragebögen inkl. aller Fragenblöcke ist in Tab. 2 dargestellt.

**Table 2:** 

Gruppe	Fragebogen zu Prämessung	Lehrveranstaltung	Modul Primärversorgung	Fragebogen zu Postmessung
	(Beginn des Moduls)			(Ende des Moduls)
	Enthaltene Fragenblöcke:			Enthaltene Fragenblöcke:
KG	Soziodemografische Angaben	Keine	Vorhanden	Subjektiv wahrgenommener Lernerfolg
	Inhaltliches Wissen			Akzeptanz der Forschungsaufgabe
	Inhaltliches Interesse			
IG	Soziodemografische Angaben	Vorhanden	Vorhanden	Subjektiv wahrgenommener Lernerfolg
	Inhaltliches Wissen			Akzeptanz der Forschungsaufgabe
	Inhaltliches Interesse			

Die Erhebungen erfolgten über Papierfragebögen

*KG* Kontrollgruppe

Im Fragenblock *„Inhaltliches Wissen“* konnten die Studierenden sich in 5 selbstentwickelten Aussagen zu ihrem schmerzmedizinischen Wissen einschätzen. Im Fragenblock *„Inhaltliches Interesse“* konnten die Studierenden sich in 5 selbstentwickelten Aussagen zu ihrem schmerzmedizinischen Interesse einschätzen. Im Fragenblock *„Subjektiver Lernerfolg“* wurde der Lernerfolg am Ende des Moduls Primärversorgung mit 9 Items erfasst. Im Fragenblock *„Akzeptanz der Forschungsaufgabe“* wurde die Akzeptanz am Ende des Moduls Primärversorgung mit 6 Items erfasst.

### Durchführung

Die Implementierung des Lehrformats *Wissenschaftliches Arbeiten im Fach Schmerzmedizin* in das Modul Primärversorgung im 5. Studienjahr wurde durch die Studienkommission der Medizinischen Fakultät Mannheim der Universität Heidelberg verabschiedet. Alle teilnehmenden Studierenden wurden über die Studienziele aufgeklärt und gaben ihre schriftliche Einwilligung. Das Lehrformat *Wissenschaftliches Arbeiten im Fach Schmerzmedizin* erfolgte für alle Studierenden des Moduls Primärversorgung verpflichtend. Die Studierenden des Lehrblocks 4 des Studienjahrs 2013/14 waren als Teilnehmer der Interventionsgruppe geplant. Sie gaben ihre schriftliche Einwilligung in die Fragebogenerhebung und nahmen an der Prä- und der Postmessung teil. Die Teilnahme an der (verpflichtenden) Lehrintervention *Wissenschaftliches Arbeiten im Fach Schmerzmedizin* sowie an der realen Versorgungsforschungsstudie *Case-Management-Programm Kreuzschmerz *lehnten sie jedoch geschlossen ab. Als Grund für die Ablehnung führten sie eine zu geringe Information über die Studienziele und das subjektive Empfinden einer Ausnutzung als unbezahlte Hilfswissenschaftler an. Ihre Vorbehalte wurden aufgegriffen und durch eine verbesserte Information über die Ziele der Lehrintervention, auch unter Einbeziehung der Fachschaft sowie der studentischen Vertreter der Studienkommission, adressiert. Das bereits durch die Studienkommission verabschiedete und hier unter Projektbeschreibung beschriebene Prozedere wurde nicht verändert. In der vorliegenden Untersuchung wurden sie als „Interventionsgruppe abgelehnt“ der Kontrollgruppe zugeordnet.

Die Prämessung erfolgte während der Einführungsveranstaltung zum Modul Primärversorgung. Im Rahmen der Einführungsveranstaltung wurde das Konzept des Moduls und in der IG auch das des Lehrformats *Wissenschaftliches Arbeiten im Fach Schmerzmedizin* den Studierenden erläutert. Offene Fragen wurden beantwortet. Die freiwillig Teilnehmenden beider Gruppen beantworteten Fragen zur Person, wie Alter, Geschlecht, Praxiserfahrungen, sowie die Fragen der in Tab. 1 aufgeführten Fragenblöcke.

Die Postmessung erfolgte am Tag der Prüfung Schmerzmedizin in der Prüfungswoche am Ende des Moduls. Alle Teilnehmer beantworteten die Fragen der in Tab. 1 aufgeführten Fragenblöcke.

Fakultätsseitig wurde die Lehrveranstaltung über den gesamten Studienzeitraum vom gleichen Dozententeam durchgeführt.

## Statistische Analysen im Rahmen der Projektevaluation

Die Analyse der Daten erfolgte mit IBM SPSS Statistics Version 24 (IBM, Armonk, New York, USA). Als deskriptive Maße wurden Häufigkeiten, Mittelwerte, Standardabweichungen und Pearson-Korrelationen berechnet. Die Überprüfung der Hypothesen erfolgte mittels varianzanalytischer Auswertungen, (einfaktorieller, „mixed-design“, „repeated-measures“) ANOVA und (multipler) Regressionsanalysen. Das Signifikanzniveau aller Analysen wurde auf α = 0,05 festgelegt.

## Ergebnisse

In einem ersten Schritt wurden die Daten einer Ausreißeranalyse unterzogen. Für alle untersuchten Variablen lagen die standardisierte Schiefe und Kurtose innerhalb der Spannweite von −3 bis +3 [12]. Die Daten wiesen keine univariaten, bivariaten oder multivariaten Ausreißer auf. In einem zweiten Schritt wurden die Voraussetzungen für varianzanalytische Verfahren überprüft. Zur Überprüfung der Normalverteilungsannahme kam der Kolmogorov-Smirnov-Test zum Einsatz, zur Überprüfung der Varianzhomogenität der Levene-Test. Die Voraussetzungen waren für alle nachstehenden Analysen erfüllt und werden daher nicht im Einzelnen berichtet.

### Hat die Teilnahme am Lehrformat *Wissenschaftliches Arbeiten im Fach Schmerzmedizin* einen Einfluss auf den subjektiv wahrgenommenen Lernerfolg?

Zunächst wurden die fünf Gruppen dichotomisiert: an der Intervention nicht teilgenommen (KG und IG abgelehnt) und an der Intervention teilgenommen (IG 1, IG 2, IG 3). Eine einfaktorielle ANOVA mit der dichotomisierten Gruppenzugehörigkeit als unabhängige Variable und dem subjektiven Lernerfolg als abhängige Variable zeigte keinen signifikanten Unterschied zwischen den beiden Gruppen (*F* (1,80) = 0,38, *p* = 0,54). Weiterhin wurden Kontraste für alle fünf Gruppen berechnet. Paarweise Vergleiche zeigten keine signifikanten Unterschiede im subjektiv wahrgenommenen Lernerfolg zwischen den einzelnen Gruppen. Tab. 3 stellt die Mittelwerte für alle 3 Interventionsgruppen dar.

**Table 3:** 

Item	IG 1 (*N* = 14)	IG 2 (*N* = 9)	IG 3 (*N* = 16)
	*M (SD)*	*M (SD)*	*M (SD)*
Ich habe mit diesem Lernmaterial viel gelernt	4,79 (0,58)	5,00 (0,71)	4,63 (0,81)
Ich glaube, dass ich nach dem Training mit dem Lernmaterial besser in der Lage bin, Situationen mit Schmerzpatienten zu beherrschen	4,71 (0,83)	5,33 (0,71)	4,63 (0,96)
Durch die Lernfälle weiß ich jetzt, worauf ich bei der Behandlung von Schmerzpatienten explizit achten muss	4,71 (0,61)	5,00 (0,71)	4,81 (0,98)
Die Lernfälle haben mich dafür sensibilisiert, Situationen genauer zu beobachten	5,14 (0,77)	5,00 (0,71)	5,19 (0,54)
Die Lernfälle haben mir eine Vorstellung davon vermittelt, wie ich mein theoretisches Wissen für mich praktisch nutzbar machen kann	4,64 (0,84)	5,00 (0,71)	4,63 (1,09)
Was ich mit der Lernumgebung gelernt habe, hätte ich ebenso gut aus einem Buch gelernt. (R)	2,71 (0,99)	2,33 (0,50)	3,31 (1,01)
Ich habe das Gefühl, durch die Arbeit mit der Lernumgebung viel gelernt zu haben	4,36 (0,75)	4,67 (0,87)	4,13 (1,09)
Ich bearbeite lieber vorgegebene Fälle, als mir selbst Szenarien auszudenken	4,21 (0,89)	4,00 (1,00)	4,13 (1,26)
Ich denke mir lieber selbst Szenarien aus und suche dazu die Theorien zusammen. (R)	3,07 (1,03)	3,22 (1,09)	2,63 (0,89)

Alle Items sind Likert-1–6-gestuft: *1* stimmt überhaupt nicht, *2* stimmt weitgehend nicht, *3* stimmt eher nicht, *4* stimmt ein wenig, *5* stimmt weitgehend, *6* stimmt genau

*M* Mittelwert, *SD* Standardabweichung, *IG* Interventionsgruppe, *R* umzupolendes Item

### Besteht ein Zusammenhang zwischen dem subjektiven Lernerfolg und der Akzeptanz der Lehrveranstaltung *Wissenschaftliches Arbeiten im Fach Schmerzmedizin*?

Eine einfaktorielle ANOVA mit der dichotomisierten Gruppenzugehörigkeit als unabhängige Variable und der Akzeptanz der Lehrveranstaltung als abhängige Variable zeigte keinen signifikanten Unterschied zwischen den beiden Gruppen (*F* (1,69) = 0,024, *p* = 0,88). Tab. 4 stellt die Mittelwerte für alle 3 Interventionsgruppen dar.

**Table 4:** 

	IG 1	IG 2	IG 3
*N*	14	9	16
*M*	22,79	23,89	23,06
*SD*	3,40	6,03	4,43

*IG* Interventionsgruppe, *M* Mittelwert, *SD* Standardabweichung

Zur Überprüfung des Zusammenhangs zwischen subjektivem Lernerfolg und Akzeptanz der Lehrveranstaltung wurden Pearson-Korrelationen für die einzelnen Gruppen sowie für Gruppencluster berechnet. In der zusammengefassten Gruppe IG 1 + IG 2 zeigte sich ein signifikanter, mittelstarker, positiver, linearer Zusammenhang zwischen dem subjektiven Lernerfolg und der Akzeptanz der Veranstaltung (*r* (23) = 0,49, *p* = 0,02). In IG 3 zeigte sich ein schwach negativer, jedoch nicht signifikanter Zusammenhang. Tab. 5 stellt die Akzeptanzmittelwerte für alle 3 Interventionsgruppen dar.

**Table 5:** 

Item	IG 1 (*N* = 14)	IG 2 (*N* = 9)	IG 3 (*N* = 16)
	*M (SD)*	*M (SD)*	*M (SD)*
Die Einbindung in ein reales wissenschaftliches Projekt hat mir Spaß gemacht	3,36 (1,45)	4,56 (1,13)	3,25 (1,13)
Die Einbindung in ein reales wissenschaftliches Projekt hat mein Verständnis über Forschungsfragen verbessert	3,29 (1,33)	3,56 (1,01)	3,44 (1,03)
Die Einbindung in ein reales wissenschaftliches Projekt im Rahmen der curricularen Lehre habe ich als Ausnutzung empfunden. (R)	3,07 (1,03)	2,89 (1,27)	2,56 (1,21)
Die für das wissenschaftliche Projekt vorgesehene Lehrzeit war zu umfangreich. (R)	2,67 (0,72)	3,56 (1,33)	3,31 (1,54)
Die für das Projekt vorgesehene Zeit war zu kurz. (R)	2,93 (0,99)	2,89 (0,93)	3,19 (1,28)
Das Gelernte lässt sich auf andere medizinische Fächer übertragen	3,79 (1,31)	4,11 (1,45)	4,44 (0,81)

Alle Items sind Likert-1–6-gestuft: *1* stimmt überhaupt nicht, *2* stimmt weitgehend nicht, *3* stimmt eher nicht, *4* stimmt ein wenig, *5* stimmt weitgehend, *6* stimmt genau

*M* Mittelwert; *SD* Standardabweichung; *IG* Interventionsgruppe, *R* umzupolendes Item

## Diskussion

### Bewertung des Lehrformats *Wissenschaftliches Arbeiten im Fach Schmerzmedizin*

Die Teilnehmerinnen und Teilnehmer dieses innovativen Lehrformats im Rahmen des Modellstudiengangs MaReCuM sollten Daten bei realen Studienpatienten der Versorgungsforschungsstudie *Case-Management-Programm Kreuzschmerz* und unter Praxisbedingungen erheben. So sollten sie im letzten Abschnitt des longitudinalen Wissenschaftscurriculums dieses Studiengangs die Gelegenheit erhalten, die erworbenen Kompetenzen unter Praxisbedingungen anzuwenden.

#### Subjektiver Lernerfolg

Am Ende des Moduls bewerteten die Studierenden der Interventionsgruppe die Aussage „Ich habe mit diesem Lernmaterial viel gelernt“ im Mittel mit „stimmt weitgehend“, die Aussage „Ich habe das Gefühl, durch die Arbeit mit der Lernumgebung viel gelernt zu haben“ mit „stimmt ein wenig“. Die Aussagen „Die Lernfälle haben mich dafür sensibilisiert, Situationen genauer zu beobachten“ sowie „Die Lernfälle haben mir eine Vorstellung davon vermittelt, wie ich mein theoretisches Wissen praktisch für mich nutzbar machen kann“ wurden ebenfalls im Mittel mit „stimmt weitgehend“ bewertet. Die Aussage „Was ich mit der Lernumgebung gelernt habe, hätte ich ebenso gut aus einem Buch gelernt“ wurde im Mittel mit „stimmt eher nicht“ bewertet. Bezogen auf den subjektiven Lernerfolg scheint die Beteiligung an einem realen Forschungsprojekt bei den Studierenden positiv bewertet worden zu sein. Der insgesamt eher geringe Lernzuwachs lässt sich durch die longitudinale Verankerung von wissenschaftlichen Inhalten im Modellstudiengang MaReCuM [13] und die Verortung der vorliegenden Lehrveranstaltung im 5. Studienjahr erklären. In diesem Ausbildungsabschnitt verfügen die Studierenden bereits über Vorkenntnisse. Darüber hinaus hatten 68 % der an der Untersuchung beteiligten Studierenden ein laufendes oder bereits abgeschlossenes Promotionsverfahren, 30 % eine Anstellung als Hilfswissenschaftler, 14 % beteiligten sich an sonstigen Forschungsprojekten und 4 % hatten ein abgeschlossenes Hochschulstudium. Eine weitere Erklärung könnte in zu umfangreich bemessenen Lernzielen liegen. Mit dem gewählten Studiendesign lässt sich die Effektivität eines theoretischen Unterrichts und eines, wie in der vorliegenden Arbeit, Unterrichts mit konkretem Bezug zu einer realen Forschungsarbeit nicht vergleichen. Ausgehend von den Ergebnissen der vorliegenden Untersuchung bietet sich hier der Ansatz für weitere Untersuchungen.

#### Akzeptanz der Lehrveranstaltung *Wissenschaftliches Arbeiten im Fach Schmerzmedizin*

Am Ende des Moduls bewerteten die Teilnehmer der Interventionsgruppe die Aussage „Die Einbindung in ein reales wissenschaftliches Projekt hat mein Verständnis über Forschungsfragen verbessert“ im Mittel mit „stimmt eher nicht“. Diese Einschätzung kann möglicherweise durch die bereits erworbenen Kompetenzen und Vorerfahrungen durch die longitudinale Verankerung von wissenschaftlichen Inhalten im Modellstudiengang MaReCuM erklärt werden. Eine Bestandsaufnahme aus dem ersten Halbjahr 2014 ergab, dass alle Lernziele der NKLM-Kapitel 6 und 14a in den Studienjahren 1–5 adressiert werden [13]. Aus dem curricularen Mapping von Eckel et al. [13] geht hervor, dass in den Studienjahren 1 und 3 Recherchetrainings mit Mitarbeitern der Bibliothek durchgeführt werden sowie eine selbstständige Literatursuche im klinischen Studienabschnitt zu verschiedenen Gelegenheiten verlangt wird. In der Vorklinik werden theoretische Grundlagen der Versuchsplanung mit kritischer Bewertung von Informationen und Quellen in den Fächern Biomathematik, medizinische Psychologie und Ethik vermittelt. Ebenfalls bereits in der Vorklinik werden in den Grundlagenfächern selbstständig Laborversuche durchgeführt und ausgewertet. Inhalte der evidenzbasierten Medizin werden dieser Erhebung zufolge verteilt über alle Studienjahre in verschiedenen Fächern vermittelt. Die Präsentation wissenschaftlicher Daten wird an mehreren Stellen des Curriculums explizit geübt, beispielsweise durch verpflichtende Referate, das Ausarbeiten von Postern sowie das Verfassen eines Abstracts. Die Autorengruppe um Eckel [13] betont den gewachsenen und in gewisser Hinsicht impliziten Charakter dieses wissenschaftlichen Curriculums im Studiengang MaReCuM. Die Studierenden der IG 2 waren darüber hinaus Teilnehmer des das Studium begleitenden Masterstudiengangs Translational Medical Research des MaReCuM. Die Nichtteilnahme an der realen Versorgungsforschungsstudie durch einige Studierende der IG kann ebenfalls Einfluss auf die Bewertung dieses Items haben. Dieser Effekt ist mit den vorliegenden Daten jedoch statistisch nicht quantifizierbar.

Die Übertragbarkeit des Gelernten auf andere medizinische Fachgebiete wurde im Mittel mit „stimmt ein wenig“ bewertet. Dieser Punkt wurde als explizites Lernziel in das Seminar der Lehrintervention *Wissenschaftliches Arbeiten im Fach Schmerzmedizin* implementiert. Möglicherweise hat der Name der Lehrintervention an dieser Stelle den Fokus zu sehr auf Schmerzmedizin gerichtet und so die intendierte Übertragbarkeit auch auf andere medizinische Fachgebiete in der Wahrnehmung der Studierenden eingeschränkt.

Der Aspekt Ausnutzung der Studierenden als unbezahlte Hilfswissenschaftler im Rahmen der curricularen Lehre wurde im Rahmen der Implementierung durch die studentischen Vertreter der Studienkommission ausgesprochen. Vonseiten der Fakultät und des Studienteams wurde dieser Aspekt explizit adressiert und der Mehraufwand durch die Implementierung der Lehrintervention transparent in Relation zum deutlich geringeren Aufwand bei Einstellung einer wissenschaftlichen Hilfskraft zur Datenerhebung im Rahmen der realen Versorgungsforschungsstudie gesetzt. Die Darstellung erfolgte unter anderem im Rahmen der Einführung in das Modul. Am Ende des Moduls bewerteten die Teilnehmer der IG die Aussage „Die Einbindung in ein reales wissenschaftliches Projekt im Rahmen der curricularen Lehre habe ich als Ausnutzung empfunden“ im Mittel mit „stimmt eher nicht“. Diese Einschätzung belegt, dass die an verschiedenen Stellen erfolgte Information und Aufklärung über die Zielsetzungen der Lehrintervention in schriftlicher und mündlicher Form wirksam war.

## Limitationen

Im ersten Durchlauf der Lehrveranstaltung konnten nicht alle in die Interventionsgruppe eingeschlossenen Studierenden auch tatsächlich an der realen Versorgungsforschungsstudie *Case-Management-Programm Kreuzschmerz* teilnehmen. Für die Nichtteilnahme an der realen Versorgungsforschungsstudie wurden verschiedene Gründe angegeben. Diese umfassten das Fehlen geeigneter Patienten in den jeweiligen Praxen, zeitliche Limitationen sowie die Weigerung einzelner Praxisinhaber, entgegen der Vereinbarung, am Projekt teilzunehmen. Dies schlägt sich in einer niedrigen Rücklaufquote der Scoresheets von 19 bis 55 % der erwartbaren Scoresheets nieder. Die Einflüsse dieser Limitation auf den subjektiven Lernerfolg sowie die Akzeptanz lassen sich nicht quantifizieren. Dennoch sind durch diese Faktoren negative Einflüsse auf Lernerfolg und Akzeptanz denkbar. Die aufgeführten Weigerungen durch die beteiligten Praxisinhaber sollten zukünftig adressiert werden. Der Zeitmangel stellt ein grundsätzliches Problem der ambulanten Patientenversorgung dar. Dies sollte bei der Auswahl zukünftiger Forschungsprojekte für diese Lehrveranstaltung berücksichtigt werden. Zur Erhebung der Effektivität der Lernzielvermittlung sollten bei zukünftigen Untersuchungen veröffentlichte und validierte Instrumente, wie das Comparative Self-Assessment (CSA; [14]), eingesetzt werden.

## Fazit für die Praxis

Die innovative und kompetenzorientierte Lehrveranstaltung konnte erfolgreich in das Curriculum des Modellstudiengangs implementiert werden. In der begleitenden Evaluation hatte die Teilnahme an der Lehrintervention *Wissenschaftliches Arbeiten im Fach Schmerzmedizin* an und für sich keinen messbaren Einfluss auf den *subjektiven* Lernerfolg. Die Lehrintervention sollte zukünftig mit den anderen, evolutionär entstandenen Lehrveranstaltungen zu einem longitudinalen Wissenschaftsmodul verknüpft und mit Blick auf die bereits vorhandenen Vorerfahrungen der Studierenden zu einem früheren Zeitpunkt abgehalten werden. So könnte sie eine Lerngelegenheit im Bereich der Arztrolle des Gelehrten darstellen und insgesamt zu einer Stärkung der wissenschaftlichen Kompetenzen beitragen. Die Implementierung der Lehrveranstaltung bietet darüber hinaus die einzigartige Gelegenheit für vergleichende Untersuchungen zum Erwerb von Wissenschaftskompetenzen sowie zum Einfluss auf die epistemologischen Überzeugungen der Studierenden im Fach Humanmedizin.
